# Survival Analysis of Tunneled Cuffed Central Venous Catheters in Maintenance Hemodialysis Patients: A Retrospective Study in China

**DOI:** 10.1155/2022/6809670

**Published:** 2022-09-17

**Authors:** Jun Hu, Guangliang Mei, Changjun Tong, Chaoqing Gao, Jing Zhang

**Affiliations:** ^1^Hemodialysis Center, Wannan Medical College Affiliated Yijishan Hospital, Wuhu 241000, China; ^2^Outpatient Office, Wannan Medical College Affiliated Yijishan Hospital, Wuhu 241000, China; ^3^Department of Nephrology, Wannan Medical College Affiliated Yijishan Hospital, Wuhu 241000, China

## Abstract

**Aim:**

The study aimed to investigate the clinical epidemiological data and the survival rate of maintenance hemodialysis patients with tunneled cuffed central venous catheters (TCCs) in a single hemodialysis center in China.

**Methods:**

We retrospectively investigated the general clinical characteristics (including sex, age, primary causes, and catheter outcome) of 316 patients undergoing maintenance hemodialysis (MHD) via TCC for >3 months at Wannan Medical College Affiliated Yijishan Hospital, Wuhu, China, from July 2011 to June 2021. The long-term survival rate of the catheters was determined by Kaplan–Meier survival analyses.

**Results:**

A total of 316 patients (137 males, 179 females) were included, with a mean age of 65.0 ± 15.5 years. The right internal jugular vein was the most commonly used central vein, accounting for 89.1% of catheterizations. After censoring for noncatheter-related events leading to the removal of the catheter, the mean survival time of the TCCs was 26.2 ± 19.8 smonths and the median survival time was 58.0 (95% CI, 54.0–62.0) months. Seventy patients had catheter loss-of-function events, with an incidence of 22.2%. Moreover, 97.3% of TCCs survived 1 year and 43.3% survived 5 years, respectively. The sex and age of the patients were not related to the survival rate (*p* > 0.05). There were also no statistical differences between the primary diseases of patients and the survival rate of TCCs (*p* > 0.05).

**Conclusion:**

In this study, we provide evidence of the mean TCC survival time beyond 2 years. We found that TCC is an effective alternative for MHD patients with poor vessel status or limited survival time or become a bridge waiting for arteriovenous fistula to mature, regardless of age, sex, and primary diseases.

## 1. Introduction

Chronic kidney disease (CKD) has become common worldwide. The prevalence of CKD in the world has reached 14.3% [1] and up to an estimated 160 million CKD in China [2], and the cost to care for patients with end-stage renal disease (ESRD) was substantial [3]. At present, hemodialysis (HD) is still the most important mode of renal replacement therapy (RRT) for ESRD patients. Worldwide, approximately 89% of dialysis patients receive HD [4]. Furthermore, it has been estimated that nearly 500,000 patients are undergoing maintenance hemodialysis (MHD) in the United States [3]. Vascular access (VA) is the lifeline of MHD patients, which mainly includes autogenous arteriovenous fistula (AVF), arteriovenous graft (AVG), and tunneled cuffed central venous catheter (TCC). However, AVG is not widely used due to its high price in China. Additionally, although NKF-KDOQI [5] guidelines suggest that autogenous AVF is the preferred VA for MHD patients, TCC is still widely used in MHD patients due to poor VA or cardiac function. In the case of TCC malfunction or failure, the HD effect will be significantly influenced, the economic burden of patients will be increased, and the lives of patients may even be threatened [6]. However, the data regarding TCC survival and related complications in MHD patients in China are limited. Based on the above findings, our retrospective study aimed to assess the characteristics and outcomes of MHD patients with TCCs and analyzed the survival and complications of TCCs in a single hemodialysis center in China.

## 2. Methods

A retrospective cohort of 316 MHD patients (137 males and 179 females) with TCCs was studied. Patients who underwent more than 3 months of MHD sessions at Wannan Medical College Affiliated Yijishan Hospital, Wuhu, China, from July 2011 to June 2021 were included. In total, 393 catheters were inserted. All the catheters were obtained from Covidien™ (Mahurkar Maxid 14.5 Fr Dual Lumen for 12 cases and Covidien Tal Palindrome™ for the rest of the cases). The right internal jugular vein (IJV) was mostly used for catheter insertion. The left IJV and femoral veins were used as alternatives for catheter insertion if the right IJV could not be used. Ultrasonic localization was performed before catheter insertion. Thereafter, the Seldinger technique was applied for the insertion of the catheter. After inserting the catheter, a chest X-ray examination was performed to assess the catheter position and complications. When the catheter position was determined, the outlet position of the catheter was adjusted so that the cuff was 2–3 cm away from the tunneled exit from the skin. After successful insertion, the venous and arterial arms were aspirated with the syringe, and heparin was used to seal the catheter. All the catheterization operations were performed by the same team of HD doctors. If the patient had never had a long-term venous catheter inserted, it was considered first cannulation. If the original cuff catheter could not be used normally or was no longer needed for various reasons, it was replaced or removed. All MHD patients underwent routine dialysis 2–3 times a week (4 hours each time). The blood flow rate of the TCCs ranged from 200–280 ml/min. Censored data included data concerning transferal to peritoneal dialysis (PD), kidney transplantation, AVF/AVG, or death due to noncatheter-related events. The final event was considered to be the removal of the catheter due to complications (thrombus, catheter-related infections, loss of function, mechanical damage, etc.). The study was approved by the Ethics Committee of Yijishan Hospital Affiliated with Wannan Medical College.

## 3. Statistical Analysis

Continuous variables were reported as means ± standard deviations. Categorical measures were presented as numbers and percentages or medians. The Kaplan–Meier method was used to analyze the survival of the catheters, and the survival curve was drawn with GraphPad Prism 5. The survival of TCCs was defined as the retention time and the number of months from catheterization to catheter removal due to catheter-related events. The survival rate between the groups was compared using the log rank test. Statistical significance was set at *p* < 0.05. Statistical analyses were performed using SPSS 16.0 software.

## 4. Results

### 4.1. Demographic Data of Patients and Reasons for Catheterization

Within this study, the catheterization of 316 patients (137 males and 179 females) with 393 catheters was analyzed. Fortunately, no patients were lost to follow-up. The mean age of the participants was 65.0 ± 15.5 years (range, 20–96 years). The median survival time of the catheters was 58 months (average working time, 26.2 ± 19.8; range, 3–90). The most common primary disease of HD is chronic glomerulonephritis, accounting for 51.6% of cases. In 316 cases, autogenous AVF could not be performed in 156 patients due to poor blood vessels (the diameter of the vein for autogenous AVF is less than 2 mm and the diameter of the vein for autogenous AVF is less than 1.5 mm). Cannulation was performed in 111 patients due to ≥2 failed autogenous AVF operations. Twenty-nine patients chose long-term catheterization due to malnutrition or prolonged maturation of the fistula. Additionally, TCCs were applied to 20 patients with advanced tumors due to the limited survival time (Table 1).

### 4.2. Main Complications of TCCs and the Reasons for Extubation

The main complications of TCC are illustrated in Table 2, including thrombosis (130/393, 33.1%), catheter-related infections (36/393, 9.2%), and superior vena cava syndrome (33/393, 8.4%). The total number of complications of TCCs was 199 (50.7%). The removal of TCCs occurred in a total of 131/316 patients: 44 after maturation of AVF or after creation of AVG, 3 after being transferred to PD, 5 after kidney transplant, 70 for catheter loss of function, and 9 for severe catheter-related infections (Table 3).

### 4.3. Catheter-Related Information and Adverse Events

The catheter-related information is depicted in Table 4. Most patients were cannulated only once; however, a small number of patients were cannulated three times. Catheters were chiefly inserted into the right IJV (350 out of 393 cases); however, the left IJV was used in 19 cases. Moreover, the right and left femoral veins were used in 16 and 8 cases, respectively. No complications occurred during cannulation due to ultrasonic localization and real-time chest X-ray examination. A total of 130 patients received thrombolytic treatment once or more, of which 9 had thrombolytic therapy more than 12 times (9/130, 6.9%), while most patients had thrombolytic therapy less than 6 times (87/130, 66.9%). In 19.2% of patients, thrombolysis was treated using urokinase only once. The catheter outcomes mainly included good function (the blood flow rate of TCCs ≥ 200 ml/min) (228/393, 58%) and adverse events, including catheter malfunction (the blood flow rate of TCCs < 200 ml/min) (147/393, 37.4%), catheter damage (11/393, 2.8%), and catheter exfoliation (7/393, 1.8%).

### 4.4. Overall TCC Survival

**Figure 1:**
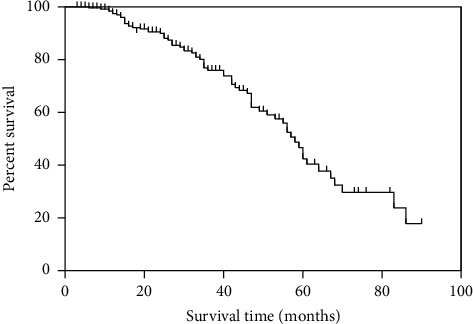
Overall tunneled cuffed central venous catheter survival of patients undergoing maintenance hemodialysis.

The longest and shortest observed catheter survival times were 96 months and 3 months, respectively. When censored for noncatheter-related events (including death due to noncatheter events, transferring to AVF/AVG, PD, or kidney transplantation) causing TCC removal (*n* = 316, 248 catheters censored, and 68 events), the 1-year and 5-year survival rates of catheters were 97.3% and 43.3%, respectively. The median survival time of the catheters was 58 months (Figure 1).

### 4.5. The Survival of TCCs According to the Distribution of Primary Diseases

**Figure 2:**
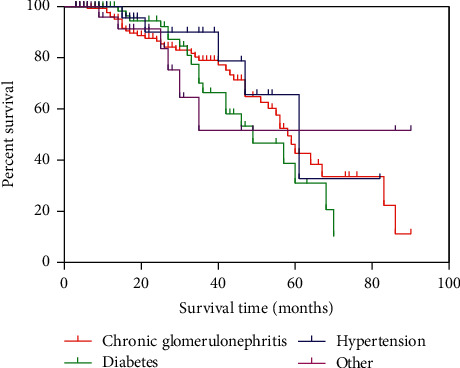
The survival of tunneled cuffed central venous catheters in different primary diseases of maintenance hemodialysis patients (*p*=0.1303).

Figure 2 shows the survival of TCCs in different primary diseases for MHD. The median survival time was 58, 49, and 61 months in chronic glomerulonephritis, diabetes, and hypertension, respectively. There were no differences in catheter survival with respect to primary diseases (*p* > 0.05).

## 5. Discussion

In this study, we retrospectively investigated the general clinical characteristics of 316 MHD patients with 393 TCCs and analyzed the long-term survival rate of TCCs using Kaplan–Meier survival analyses. The data from the Yijishan Hospital hemodialysis center shows that the mean survival time of TCCs is approximately 2 years, regardless of age, sex, and the distribution of primary diseases in MHD patients.

In a cohort of 5,466 patients, the survival rate at 5 years was 48% in central venous catheter (CVC) patients, including TCCs and non-tunneled catheters [7]. A recent study reported that the survival of CVC-Tuff in 1 ear was 93.59% [8]. Our data showed that the 1-year and 5-year survival rates of TCCs were 97.3% and 43.3%, respectively, which is consistent with results of the above two studies [7, 8]. Min et al. [9] showed a median survival time of 45.0 (95% CI, 29.3–69.7) months for TCCs, which differs from our data (median survival time: 58 months). Although both studies included Chinese patients, the following differences were present: (1) different sample size (59 patients and 316 patients); (2) in the study by Min et al. [9], the average age of HD patients was 68.6 ± 14.6 years and diabetes was observed in 35.6% of the patients, whereas our data showed an average age of 65.0 ± 15.5 years, with diabetes in 25.3% of the patients. Therefore, Shi et al. found that advanced age and diabetes mellitus (DM) were significant risk factors for TCC failure, while our data showed that age and distribution of the primary diseases in MHD patients were not related to TCC survival. After censoring for noncatheter-related events that led to catheter removal, the Kaplan–Meier survival analysis calculated an average TCC survival time of 26 months, which is slightly higher than that of the previous study, which reported a mean survival time of 615 days [10].

The KDOQI 2019 guidelines [11] suggested that when CVCs need to be used and the use time is expected to exceed 3 months, the CVC placement sequence is as follows: internal jugular, external jugular, femoral, and subclavian. Santos-Ontiveros et al. [8] showed that the right jugular vein was the most commonly used for catheterization. In our study, the right internal jugular vein was predominantly used. According to the order of use frequency of VA site from high to low, they are right internal jugular vein, left internal jugular vein, right femoral vein, and left femoral vein. However, the external jugular and subclavian veins were not used in our study.

We found that thrombosis, infection, and superior vena cava syndrome were still the main complications of TCCs, which is similar to the results of the previous study that reported TCC-related complications of infection, thrombosis, and central vein stenosis [12]. Superior vena cava syndrome is a clinical manifestation of central vein stenosis. However, not all patients with central vein stenosis were examined by digital subtraction angiography. Therefore, we could only diagnose superior vena cava syndrome according to the clinical symptoms and signs. The incidences of TCC infection in the two studies were 26% (29/109) [13] and 20.3% (16/79) [14], respectively, which are much higher than those of our data (9.2%). The reason for this may be that the percentage of patients with diabetes in our study is relatively low (only accounting for 25.3%), and diabetic patients are more prone to infection, including catheter-related infection. In a study by Zanoni et al. [15], 33/413 (8.2%) patients were diagnosed with catheter-related bloodstream infections (CRBSIs), and diabetic patients accounted for 88/413 (21%), which is similar to our data. It was estimated that 70% of access-related bloodstream infections were found in patients with TCCs [16]. To effectively reduce the TCC-related bloodstream infection incidence rate, the Centers for Disease Control and Prevention (CDC) recommended several core interventions, including good hand hygiene practices, catheter care observations, education for both patients and staff on catheter care, chlorhexidine for skin antisepsis, catheter hub disinfection, and antimicrobial ointment application during dressing change [17]. The main risk factors for bloodstream infections in TCCs are the indwelling time of the catheter [18], previous catheter-related bacteremia [19], left IJV catheters [20], hypoalbuminemia, and immunosuppression. Although there were some risk factors in our study, such as left-sided IJV catheters and long catheterization time, the incidence of catheter-related infection was only 9.2%. The Infectious Diseases Society of America (IDSA) guidelines recommend catheter removal when the following conditions occur: metastatic infections, severe sepsis, specific virulent organisms, or less virulent but difficult to eradicate organisms [21]. In our study, the incidence of TCC removal due to severe infection was only 2.3%; this reflects the clinical and nursing advantages in our center.

Our data showed that the incidence of catheter-related thrombosis was 33.1%, similar to a study by Shrestha et al. [22], which showed an incidence of 27.18%. Peng et al. [23] reported that thrombosis is most likely to occur at the site where most of the blood flow has lost spiral rotation due to TCC insertion, as well as geometric factors, including catheter shape, diameter, and length,which disturb the flow in the central veins and cause thrombus formation in the central vein. Additionally, thrombosis formation in the catheter could result in catheter malfunction and loss of catheter function. This is because thrombosis formation in the catheter impairs the patency of the catheter lumen and leads to poor catheter function, even catheter loss of function [24]. In our study, 45/393 (11.5%) catheters were removed due to thrombosis-related catheter loss of function. DM is considered a significant prothrombotic state [25] and therefore increases the risk for recurrent thrombosis (HR, 3.19 (1.09–9.41); *p*=0.03). Furthermore, DM significantly increases the risk of recurrent catheter-related thrombosis and consequently decreases 1-year catheter survival [26].

Catheter service time increases with age, which is a protective factor [27]. Compared with patients younger than 60 years, patients aged 70–79 and those ≥80 years experienced lower rates of CVC complications [28]. However, contrary to these previous studies [27, 28], our study showed that in 316 MHD patients with TCCs, age was not associated with TCC survival.

## 6. Limitations

There were several limitations in our study. First, we could not exclude the selective bias of patients, including age, sex, and primary diseases of patients. Second, the distribution of each primary disease is different due to the limited sample size and uneven distribution. However, since our study was retrospective, bias may have occurred due to the significant difference in the numbers of various primary diseases. Expanding the sample size may reduce this bias. Lastly, the population of this single hemodialysis center in China may not be representative of populations in other countries. Further prospective studies should be performed to evaluate the related factors and risk factors affecting the catheter survival time in MHD patients.

## 7. Conclusion

Our study findings provide evidence of the mean TCC survival time beyond 2 years. We found that TCC is an effective alternative for MHD patients with poor vessel status or limited survival time, or become a bridge waiting for internal fistula to mature, regardless of age, sex, and primary disease.

## Figures and Tables

**Table 1:** Demographics and reasons for catheterization.

Variable	Patients (*N* = 316)
Age (range)	65.0 ± 15.5 (20–96)
Sex	
Male	137 (43.3%)
Female	179 (56.7%)

Primary disease	
Hypertension	37 (11.7%)
Diabetes	80 (25.3%)
Chronic glomerulonephritis	163 (51.6%)
Others	36 (11.4%)

HD vintage, months	48.7 ± 35.0 (4–244)
Average survival time of TCC, months	26.2 ± 19.8 (3–90)
Median survival time of TCC, months	58.0
Male	56.0
Female	59.0
>65 years	58.0
≤65 years	57.0

Reason for catheterization	
No access options	156 (49.4%)
Failed AVF	111 (35.1%)
Immature AVF	29 (9.2%)
Advanced cancer patients	20 (6.3%)

Continuous variables are expressed as the mean ± standard error of the mean and categorical variables are summarized as *n* (%). HD, hemodialysis; TCC, tunneled cuffed central venous catheter; AVF, autogenous arteriovenous fistula.

**Table 2:** The main complications of TCCs.

Main complications	TCC (*N* = 393)
Thrombus	130 (33.1%)
Catheter-related infections	36 (9.2%)
Superior vena cava syndrome	33 (8.4%)
Total	199 (50.7%)

Data are expressed as *n* (%). TCC, tunneled cuffed central venous catheter.

**Table 3:** The reasons for extubation.

Reasons for extubation	TCC (*N* = 393)
Transfer to AVF/AVG	44 (11.2%)
Transfer to PD	3 (0.8%)
Transfer to kidney transplant	5 (1.3%)
Catheter loss of function	70 (17.8%)
Due to thrombus	45 (11.5%)
Due to nonthrombus	25 (6.4%)
Catheter-related infections	9 (2.3%)
Total	131

Data are expressed as n (%). TCC, tunneled cuffed central venous catheter; AVF/AVG, autogenous arteriovenous fistula/arteriovenous graft; PD, peritoneal dialysis.

**Table 4:** Catheter-related information and adverse events.

Variable	Patients (*N* = 316)
Number of catheterizations	
1	260 (82.3%)
2	35 (11.1%)
3	21 (6.6%)

The cumulative number of catheterizations	393
Categorized VA site	Catheters (*N* = 393)
Right internal jugular vein	350 (89.1%)
Left internal jugular vein	19 (4.8%)
Right femoral vein	16 (4.1%)
Left femoral vein	8 (2.0%)

Number of thrombolytic treatments	Catheters (*N* = 393)
1	25 (19.2%)
2	19 (14.6%)
3	21 (16.2%)
4	11 (8.5%)
5	11 (8.5%)
6	7 (5.4%)
7	7 (5.4%)
8	5 (3.8%)
9	7 (5.4%)
10	3 (2.3%)
11	1 (0.8%)
12	4 (3.1%)
≥13	9 (6.9%)

Total	130
Catheter outcome	Catheters (*N* = 393)
Catheter with good function	228 (58.0%)
Catheter malfunction	147 (37.4%)
Catheter damage	11 (2.8%)
Catheter exfoliation	7 (1.8%)

Data are expressed as *n* (%). VA, vascular access.

## Data Availability

The data used to support the findings of this study are available from the corresponding author upon reasonable request.

## Additional Points

*What is known.* The mean TCC survival time is beyond 2 years. Vascular access is the lifeline of maintenance hemodialysis (MHD) patients. Autogenous arteriovenous fistula (AVF) is the first choice for MHD patients. Tunneled cuffed central venous catheters (TCC) are an alternative to AVF. *What is new.* TCC is an effective alternative for MHD patients with poor vessel status or limited survival time or becomes a bridge waiting for an internal fistula to mature, regardless of age, sex, and primary diseases.
